# Incidence and survival of primary metastatic breast cancer in Denmark: implication of breast cancer screening, classification, and staging practice

**DOI:** 10.2340/1651-226X.2023.37270

**Published:** 2024-05-06

**Authors:** Tobias Berg, Maj-Britt Jensen, Maria Rossing, Christian T. Axelsen, Iben Kümler, Lise Søndergaard, Marianne Vogsen, Ann S. Knoop, Bent Ejlertsen

**Affiliations:** a Danish Breast Cancer Group, Department of Oncology, Copenhagen University Hospital, Rigshospitalet, Denmark; b Department of Oncology, Copenhagen University Hospital, Rigshospitalet, Denmark; c Center for Genomic Medicine, Copenhagen University Hospital, Rigshospitalet, Denmark; d Department of Clinical Medicine, Faculty of Health and Medical Sciences, University of Copenhagen, Copenhagen, Denmark; e Department of Oncology, Aarhus University Hospital, Aarhus, Denmark; f Department of Oncology, Copenhagen University Hospital, Herlev Hospital, Herlev, Denmark; g Department of Oncology, Zealand University Hospital, Næstved Sygehus, Denmark; h Department of Oncology, Odense University Hospital, Odense, Denmark; i Department of Clinical Research, University of Southern Denmark, Odense, Denmark

**Keywords:** Breast cancer, metastatic, epidemiology

## Abstract

**Background:**

Primary metastatic breast cancer (pMBC) accounts for 5–10% of annual breast cancers with a median survival of 3–4 years, varying among subtypes. In Denmark, the incidence of breast cancer increased until 2010, followed by a stabilisation. Several factors influencing pMBC incidence and survival, including screening prevalence, staging methods, and classification standards, remain pivotal but inadequately documented.

**Material and method:**

This retrospective observational study involving pMBC patients diagnosed between 2000 and 2020 encompassed all Danish oncology departments. Data from the Danish Breast Cancer Group database and the National Patient Register included diagnosis specifics, demographics, treatment, and follow-up.

**Results:**

Between 2000 and 2020, 3,272 patients were diagnosed with pMBC, a rise from 355 patients in 2000–2004 to 1,323 patients in 2015–2020. The increase was particularly observed in patients aged 70 years or older. Changes in tumour subtypes were observed, notably with a rise in human epidermal growth factor receptor 2 (HER2)-positive cases but a steady distribution of estrogen receptor (ER) status. Diagnostic practices changed over the two decades, with 6% evaluated with PET/CT (positron emission tomography–computed tomography) or CT (computed tomography) with a bone evaluation in 2000–2004 and 65% in 2015–2020. Overall survival (OS) improved from 23 months in 2000–2004 to 33 months in 2015–2020. In patients with ER-positive and HER2-positive disease, the multivariable model showed improved survival by year of diagnosis, and further, patients with ER-negative/HER2-negative disease fared worse the first 2 years after diagnosis.

**Interpretation:**

Our study delineates changes in the treatment and survival of pMBC over two decades. Stage migration, screening introduction, and changes in registration practice, however, prevent a valid assessment of a possible causal relationship.

## Introduction

Approximately 5–10% of annual breast cancer patients are diagnosed with synchronous distant metastases, primary metastatic breast cancer (pMBC), an essentially non-curative form of breast cancer mainly treated with palliative intentions and an expected median survival of 3–4 years with a difference between subtypes [1, 2].

In Denmark, the incidence of breast cancer increased monotonically among women up to 2010, where the rate of increase levelled off [3]. Several factors, however, are essential to understand stage-specific incidence and survival, including prevalence of screening, use of staging tests, classification, and coding of breast cancer. Firstly, population-based breast cancer screening for women aged 50 to 69 years, was introduced stepwise in Denmark. Three counties introduced breast cancer screening between 1991 and 1994, corresponding to approximately 20% of Danish women in this age group, and nationwide screening was effectuated between 2007 and 2010 [4]. Secondly, staging has changed, and compliance with Danish guidelines concerning staging has not been documented. Clinical examination, X-ray of the chest, and bone scintigraphy or X-ray of the axial skeleton were introduced for the detection of operable breast cancer with the establishment of the Danish Breast Cancer Group (DBCG) in 1977 [5]. Staging was modified in 1990 to only require bone scintigraphy in case of bone pain or abnormal biochemical tests and in 2022 to only require chest X-ray for pulmonary symptoms [6, 7]. Since January 2023, PET-CT has been recommended for staging patients with locally advanced disease [8, 9]. Lastly, postoperative classification and coding of operable breast cancer (pT/pN) have been registered in the DBCG database since 1978 in combination with the presence or absence of distant metastases while clinical stage (cT/cN) has not. By registering pathological tumour size, number of excised and positive lymph nodes, some, but not all, flaws introduced by changes in classification practice have been avoided. Neoadjuvant chemotherapy was initially reserved for surgically inoperable patients, extended to include larger tumours (>5 cm in 2010), and by 2016 encompassing most estrogen receptor (ER)-negative and/or human epidermal growth factor receptor 2 (HER2)-positive tumours. With greater use of neoadjuvant therapy, clinical staging and registration expanded. These changes, particularly for larger tumours, have led to some being categorised as both clinically operable and locally advanced over time. Furthermore, an inconsistency is seen concerning the classification of lymph node metastases (cN3 or pN3) as curable or non-curable after changes in the American Joint Committee on Cancer (AJCC) [10, 11].

Compared with Australia, Canada, Norway, Sweden, and the United Kingdom (UK), an adverse stage distribution was observed during 2000–2007 in Denmark [12]. The proportion with stage I was 30.1% in Denmark compared with 42–45% in the other countries. The proportion of stage III–IV ranged from 22% in Denmark to 8% in Sweden. The largest international differences in survival were found among older women or those who were not staged, and these two groups had a low survival in the UK and Denmark which indicate that women in the UK and Denmark may more often be incompletely staged than women in other countries, particularly if frail or older. Incomplete staging may lead to uncertainty about treatment that otherwise would have been recommended by guidelines [13, 14]. Treatment recommendations in modern times do not stratify based on age alone, despite elderly patients rarely being included in clinical trials and often having more complex co-morbidities [15–18]. Moreover, treatment recommendations and options have significantly changed over the past 20 years. For ER-negative disease, due to an increase in adjuvant chemotherapy, there has been a shift from anthracycline and taxane-containing chemotherapy to other agents and, most recently, to immunotherapy. Patients with HER2-positive disease have witnessed the introduction of HER2-targeted agents alongside an increased emphasis on continuous HER2 inhibition complemented by several new drugs beyond trastuzumab. Lastly, there has been a noticeable de-escalation for patients with ER-positive disease, particularly in first-line treatments, towards a greater reliance on endocrine therapy and, lately, cyclin-dependent kinase (CDK)4/6 inhibitors [18–20].

A lack of survival improvement among patients diagnosed with pMBC was seen in a DBCG study covering all patients from 1995 to 2012 [21]. In this study, we evaluated the changes in prognosis for Danish patients diagnosed with pMBC from 2000 to 2020 according to calendar year, screening, staging, registration practice, and treatment.

## Method and patients

This was a retrospective, observational study involving all Danish Departments of Oncology (12 sites).

### Study population

The DBCG database covers patients with pMBC since 2000 and recurrent metastatic breast cancer since 2005. Patients with a first-time invasive breast cancer are included with >95% completeness [22]. Primary metastatic breast cancer was defined as evidence of distant metastases within 90 days of diagnosis of invasive breast cancer. We included all women aged 18 years or above, diagnosed with pMBC between 1st of January 2000 and 31st of December 2020.

### Data sources

Data on diagnosis, pathology, demographics, treatment, and follow-up were collected from the DBCG database. The following data were obtained: date of diagnosis, treatment type, date of death, localisation of metastases, ER/HER2 status. The Danish Civil Registration System was linked to the DBCG by personal identification number to secure complete follow-up on vital status and emigration until 1st of June 2023. Data on radiological examinations and information from surgeries, radiotherapy, and comorbidities were retrieved from the National Patient Register (NPR). The historical screening cohorts in Copenhagen (1990–2007) and Fyn (1993–2007) was obtained from the Danish National Archives and the Danish Quality Database for Breast Cancer Screening for 2007 and onwards for all of Denmark.

### Statistics

Categorical variables were described by counts and proportions. Age was described by median and interquartile range (IQR). χ^2^-test, and Fisher’s exact test were used to compare baseline characteristics for categorical variables and treatments in first-line, excluding unknowns. Confidence intervals (CIs) for age distributions within each year were calculated. Median overall survival (OS) is reported together with survival measures according to time after diagnosis using the Kaplan-Meier method. Overall survival was defined as the interval between the diagnosis and death from any cause. Median follow-up was calculated by the reverse Kaplan-Meier method [23].

Univariable and multivariable Cox proportional hazard regression models were applied to assess the hazard of death (HR), and corresponding CIs and statistical significance were assessed by the log-rank test. Factors presented in Table 4 were considered for multivariable analyses. Factors found significant in a univariable model (*p* < 0.1) were included in the multivariable analysis and were: year of diagnosis (continuous), age at diagnosis (continuous with 5-year increments), visceral disease (yes or no), Charlson Comorbidity Index (CCI) (0, 1–2 or 3) and immunohistochemistry (IHC) subtype (ER-pos/HER2-neg, double-negative breast cancer [DNBC], HER2-positive, unknown). Interaction was evaluated between the year of diagnosis and subtype by applying the Wald test in the multivariable models. The proportional hazards assumption was tested using the Schoenfeld residuals and by including time-dependent variables in the model. For age, HER2-positive, CCI:3, and visceral disease, we included a log-time dependency. For DNBC, we examined years 0–2 and 2+, and for IHC unknown, we examined years 0–1 and 1+ (data not shown).

### Definitions

Comorbidity was described according to the CCI and based on hospital contacts using ICD-8 and ICD-10 data up to 10 years before the date of diagnosis [24].

Screen-detected tumours were defined as tumours identified within 60 days after a screen-participation. Interval tumours were tumours diagnosed between 60 days and 2.5 years after a screen participation. All other tumours for women aged 50–69 years were deemed ‘Not screen related’.

Diagnostic workup was grouped as F-18 fluorodeoxyglucose (FDG)-PET/CT or CT with a bone evaluation (bone scintigraphy or MRI of the spine); CT scan; bone scintigraphy with or without ultrasound liver; other scans (e.g. X-ray); and unknown. This grouping was chosen to reflect current European Society for Medical Oncology (ESMO) and National Comprehensive Cancer Network (NCCN) guidelines, historical recommendations, and insufficient staging [25, 26]. Patients were only counted once within each treatment category in Table 2. Indication for radiotherapy, RT (if not registered in the DBCG database) was based on the number of treatment fractions, except for stereotactic radiotherapy (SRT), which has a separate code in NPR. One to 10 fractions were classified as RT with palliative intent, 15–35 fractions as RT to the breast and 11–14 or 35+ fractions as unknown intent and organ. Double-negative breast cancer was used as progesterone receptor analysis is not a standard pathological examination in Denmark, and true triple-negative IHC could not be confirmed.

**Table 1:** Baseline characteristics for all patients diagnosed with primary metastatic breast cancer 2000–2020 overall and by time-period.

Characteristic	Overall, *N* = 3,272		2000–2004, *N* = 355		2005–2009, *N* = 676		2010–2014, *N* = 918		2015–2020, *N* = 1,323		*P*
	*n*	%	*n*	%	*n*	%	*n*	%	*n*	%	
**Age, median (IQR)**	68 (57, 77)		64 (56, 72)		67 (56, 75)		69 (58, 78)		71 (58, 78)		
**Age Group**											<0.001
<40	122	3.8	13	3.7	27	4.0	30	3.3	52	4.0	
40–49	318	9.7	37	10	60	8.9	93	10	128	9.7	
50–59	540	17	79	22	136	20	136	15	189	14	
60–69	806	25	116	33	190	28	234	25	266	20	
70–79	935	29	84	24	173	26	246	27	432	33	
80+	551	17	26	7.3	90	13	179	19	256	19	
**ER status**											0.40
Negative	675	21	69	20	125	19	195	21	286	22	
Positive	2,584	79	279	80	546	81	722	79	1,037	78	
Unknown	13	0	7	0	5	0	1	0	0	0	
**HER2 status**											<0.001
Negative	2,228	68	128	36	420	62	671	73	1,009	76	
Positive	719	22	67	19	159	24	197	22	296	23	
Unknown	325	10	160	45	97	14	50	5	18	1	
**IHC subtype**											0.004
DNBC	337	10	25	7	58	9	100	11	154	12	
ER-pos/HER2-neg	1,890	58	103	29	361	53	571	62	855	65	
HER2-pos	719	22	67	19	159	24	197	22	296	22	
Unknown	326	10	160	45	98	14	50	5	18	1	
**Metastatic locations**											
Bone	2,196	67	204	57	436	64	622	68	934	71	<0.001
Liver	837	26	76	21	167	25	267	29	327	25	0.02
CNS	107	3.3	8	2.3	33	4.9	24	2.6	42	3.2	0.04
Lung	1,137	35	118	33	252	37	320	35	447	34	0.40
Visceral disease	1,799	55	181	51	382	57	521	57	715	54	0.20
Bone-only	871	27	114	32	166	25	226	25	365	28	0.02
**Screen participation**											<0.001
Screen-detected	41	1.3	0	0	5	0.7	13	1.4	23	1.7	
Interval cancer	88	2.7	0	0	9	1.3	34	3.7	45	3.6	
Not screen-related	1,217	37	195	55	312	46	323	35	387	29	
Not screen candidate	1,926	59	160	45	350	52	548	60	868	65	
**CCI**											0.07
0	2,379	73	275	77	514	76	642	70	948	72	
1	474	14	42	12	87	13	152	17	193	15	
2	242	7.4	26	7.3	43	6.4	66	7.2	107	8.1	
3+	177	5.4	12	3.4	32	4.7	58	6.3	75	5.7	
**Radiological exam**											
CT thorax	2,124	65	75	21	422	62	716	78	911	69	<0.001
CT abdomen	2,078	63	67	19	391	58	709	77	911	69	<0.001
CT brain	306	9.3	16	4.5	77	11	91	9.9	122	9.2	0.004
MRI brain	228	7.0	6	1.7	50	7.4	66	7.2	106	8.0	<0.001
MRI spine	818	25	27	7.6	174	26	244	27	373	28	<0.001
Chest X-ray	2,239	68	253	71	530	78	651	71	805	61	<0.001
Bone scintigraphy	559	17	34	9.6	234	35	164	18	127	9.6	<0.001
FDG-PET/CT	810	25	0	0	52	7.7	195	21	563	43	<0.001
Ultrasound liver	693	21	110	31	187	28	181	20	215	16	<0.001

DNBC: double-negative breast cancer; CCI: Charlson Comorbidity Index; ER: estrogen receptor; HER2: human epidermal growth factor receptor 2; IHC: immunohistochemistry.

**Table 2:** Treatment patterns in first-line for primary metastatic breast cancer patients by IHC subtype and by time-period.

Treatments	2000–2004			2005–2009			2010–2014			2015–2020		
	DNBC, *N* = 25	ER-pos/HER2-neg, *N* = 103	HER2-pos, *N* = 67	DNBC, *N* = 58	ER-pos/HER2-neg, *N* = 361	HER2-pos, *N* = 159	DNBC, *N* = 100	ER-pos/HER2-neg, *N* = 571	HER2-pos, *N* = 197	DNBC, *N* = 154	ER-pos/HER2-neg, *N* = 855	HER2-pos, *N* = 296
**Chemotherapy**												
Epirubicin	15	30	36	18	60	24	21	70	8	26	38	7
	(60%)	(29%)	(54%)	(31%)	(17%)	(15%)	(21%)	(12%)	(4.1%)	(16%)	(4.6%)	(2.4%)
Taxane	2	7	4	19	60	41	30	76	37	52	66	38
	(8.0%)	(6.8%)	(6.0%)	(33%)	(17%)	(26%)	(30%)	(13%)	(19%)	(35%)	(7.7%)	(13%)
Other	2	1	5	6	9	36	17	19	96	30	20	173
	(8.0%)	(1.05%)	(7.5%)	(10%)	(2.5%)	(23%)	(17%)	(3.3%)	(49%)	(19%)	(2.3%)	(58%)
No CT	6	65	22	15	232	58	32	406	56	46	731	78
	(24%)	(63%)	(33%)	(26%)	(64%)	(36%)	(32%)	(71%)	(28%)	(29%)	(85%)	(26%)
**Endocrine therapy**												
Endocrine treatment	3	84	25	5	291	60	12	474	62	17	769	92
	(12%)	(82%)	(37%)	(8.6%)	(81%)	(38%)	(12%)	(83%)	(31%)	(11%)	(90%)	(31%)
No ET	22	19	42	53	70	99	88	97	135	137	86	204
	(88%)	(18%)	(63%)	(91%)	(19%)	(62%)	(88%)	(17%)	(69%)	(89%)	(10%)	(69%)
**HER2-targeted treatment**												
HER2 targeted therapy	0	0	14	1	2	95	0	4	150	0	6	232
	(0%)	(0%)	(21%)	(1.7%)	(0.6%)	(60%)	(0%)	(0.7%)	(76%)	(0%)	(0.7%)	(78%)
No HER2	25	103	53	57	359	64	100	567	47	154	849	64
	(100%)	(100%)	(79%)	(98%)	(99%)	(40%)	(100%)	(99%)	(24%)	(100%)	(99%)	(22%)
**Surgery**												
Lumpectomy	3	10	6	6	29	12	5	52	12	19	47	11
	(12%)	(9.7%)	(9.0%)	(10%)	(8.0%)	(7.5%)	(5.0%)	(9.1%)	(6.1%)	(12%)	(5.5%)	(3.7%)
Mastectomy	7	42	22	11	56	36	17	78	40	23	67	40
	(28%)	(41%)	(33%)	(19%)	(16%)	(23%)	(17%)	(14%)	(20%)	(15%)	(7.8%)	(14%)
No surgery	15	51	39	41	276	111	78	441	145	112	741	245
	(60%)	(50%)	(58%)	(71%)	(76%)	(70%)	(78%)	(77%)	(74%)	(73%)	(87%)	(83%)
**Radiotherapy**												
Breast	3	7	4	4	10	7	5	24	3	15	27	12
	(12%)	(6.8%)	(6.0%)	(6.9%)	(2.8%)	(4.4%)	(5.0%)	(4.2%)	(1.5%)	(9.7%)	(3.2%)	(4.1%)
Palliative	7	20	16	8	70	40	13	121	25	27	161	48
	(28%)	(19%)	(24%)	(14%)	(19%)	(25%)	(13%)	(21%)	(13%)	(18%)	(19%)	(16%)
SRT	0	2	0	0	1	1	2	2	2	1	7	3
	(0%)	(1.9%)	(0%)	(0%)	(0.3%)	(0.6%)	(2.0%)	(0.4%)	(1.0%)	(0.6%)	(0.8%)	(1.0%)
Unknown indication	0	3	1	6	6	9	6	37	18	16	57	24
	(0%)	(2.9%)	(1.5%)	(10%)	(1.7%)	(5.7%)	(6.0%)	(6.5%)	(9.1%)	(10%)	(6.7%)	(8.1%)
No RT	15	71	46	40	274	102	74	387	149	95	603	209
	(60%)	(69%)	(69%)	(69%)	(76%)	(64%)	(74%)	(68%)	(76%)	(62%)	(71%)	(71%)

326 patients were excluded due to missing HER2 or ER. DNBC: Double-negative breast cancer; CT: chemotherapy; ET: endocrine therapy; SRT: Stereotactic radiotherapy; ER: Estrogen receptor; HER2: human epidermal growth factor receptor 2; IHC: immunohistochemistry.

## Results

**Figure 1:**
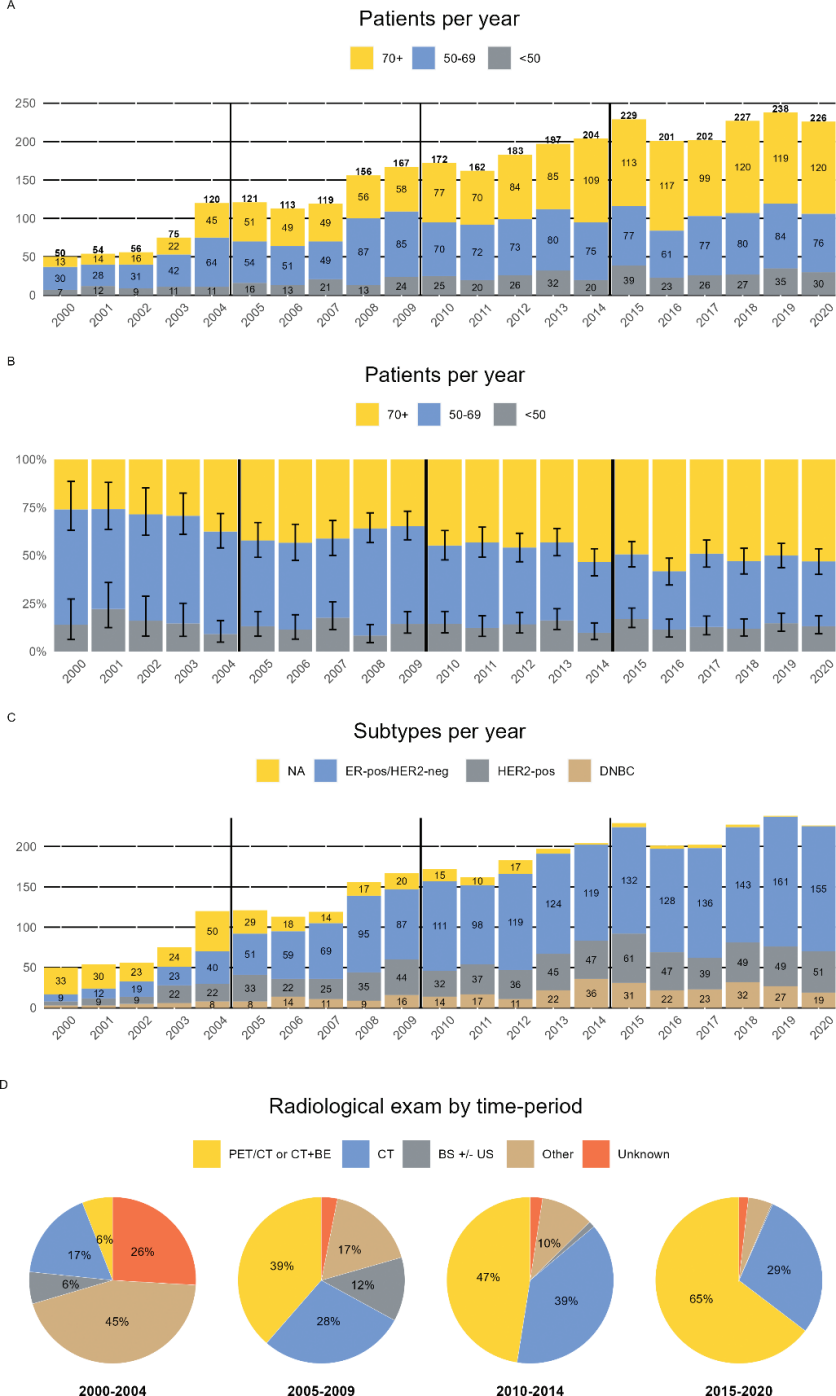
The distribution of patients with primary metastatic breast cancer per. (A) year and age group, (B) year (relative), (C) year and subtype and (D) radiological examinations by time period. DNBC: Double-negative breast cancer; BE: Bone-evaluation (bone scintigraphy or MRI of the spine); BS: Bone-scintigraphy; US: ultrasound liver; ER: estrogen receptor; HER2: human epidermal growth factor receptor 2.

Between the 1st of January 2000 and 31st of December 2020, 90.812 women were diagnosed with invasive breast cancer of which 3272 (3.6%) had pMBC. Categorised by time periods, we saw a rise from 355 patients in 2000–2004 to 1,323 patients in 2015–2020 (Table 1, Figure 1A). The overall median age was 68 years (IQR; 57–77). The increase in absolute numbers was lowest for patients younger than 50 years and highest for patients aged 70 years or above (Figure 1A), whereas the relative increase was smallest for patients aged 50–69 years and highest for patients 70 years or older. This is also reflected in Figure 1B; in year 2000 the distribution was 14% (95% CI; 6–27%), 60% (95% CI; 45–73%) and 26% (95% CI; 15–40%) for the age groups <50 years, 50–69 years , ≥70 years, respectively, and in 2020 the numbers were 13% (95% CI; 9–18%), 33% (95% CI; 27–40%) and 53% (95% CI; 46–60%). Only 41 tumours were identified by screening and 88 were determined as interval cancers. Breast cancer screening was between 2000 and 2007 and was restricted to less than 20% of women aged 50–69 years, and screening was fully implemented for this age group by 2010. As the age distributions changed over years, the incidence per 100.000 women was 0.4 for <50 years, 4.9 for 50–69 years and 3.8 for 70+ years in 2000, and 1.7, 10.3 and 26.0 in 2020, respectively. We present these numbers to ascertain that our change in distribution was not due to demographic shifts alone [27].

### Staging

Until 2004, FDG-PET/CT was not used for staging, after which its use gradually increased from 7.7% in 2005–2009 to 43% of patients in 2015–2020 (Table 1). The use of CT of the chest, abdomen, and pelvis peaked in 2010–2014 (Table 1), and CT was increasingly combined with a bone evaluation (bone scintigraphy or MRI of the spine). Overall, the proportion with stage determined by FDG-PET/CT or CT with bone evaluation increased from 6 to 65% (Figure 1D). Other imaging examinations, e.g. bone scintigraphy and abdominal ultrasound, were, to a limited extent, used periodically.

### Classification

Ten per cent were ER-negative/HER2-normal that is double-negative (DNBC), 22% were HER2-positive and 58% were ER-positive/HER2-negative. However, 326 patients (10%) could not be allocated an IHC subtype, mainly due to missing HER2-status (325 patients) (Figure 1C). A significant difference was seen in subtype distribution over the years, but this result was hampered by the high number of missing HER2 for patients between 2000 and 2010. For the individual receptors there was no change in ER-status but an overall change in HER2-status, again driven by unknowns (*p* < 0.001). Subtypes differed per age, with a higher representation DNBC and HER2-positive tumours among patients below 40 years; however, most patients with unknown HER2-status were over 70 years (Supplementary Tables S1 and S2).

### Treatment

First-line treatment varied from 2000 to 2020 (Table 2). For patients with ER-positive, HER2-negative disease, we saw a steady decrease in the use of chemotherapy from 37% in 2000–2004 to 15% in 2015–2020 (*p* ≤ 0.001). This was accompanied by an increase in endocrine treatment from 82 to 90% in 2015–2020 (*p* = 0.02). Among DNBC a shift in chemotherapy preferences was evident, as the use of epirubicine and taxanes decreased from 68 to 51% (*p* = 0.16). HER2-targeted treatment rose for HER2-positive disease from 21% in 2000–2004 to 78% in 2015–2020 (*p* < 0.001). Surgery within a year after diagnosis saw a major decrease with 48, 26, 23, and 15% for each time period (*p* < 0.001). Radiotherapy of the breast was seen in 3–7% of patients with no substantial change over time.

### Survival

**Figure 2:**
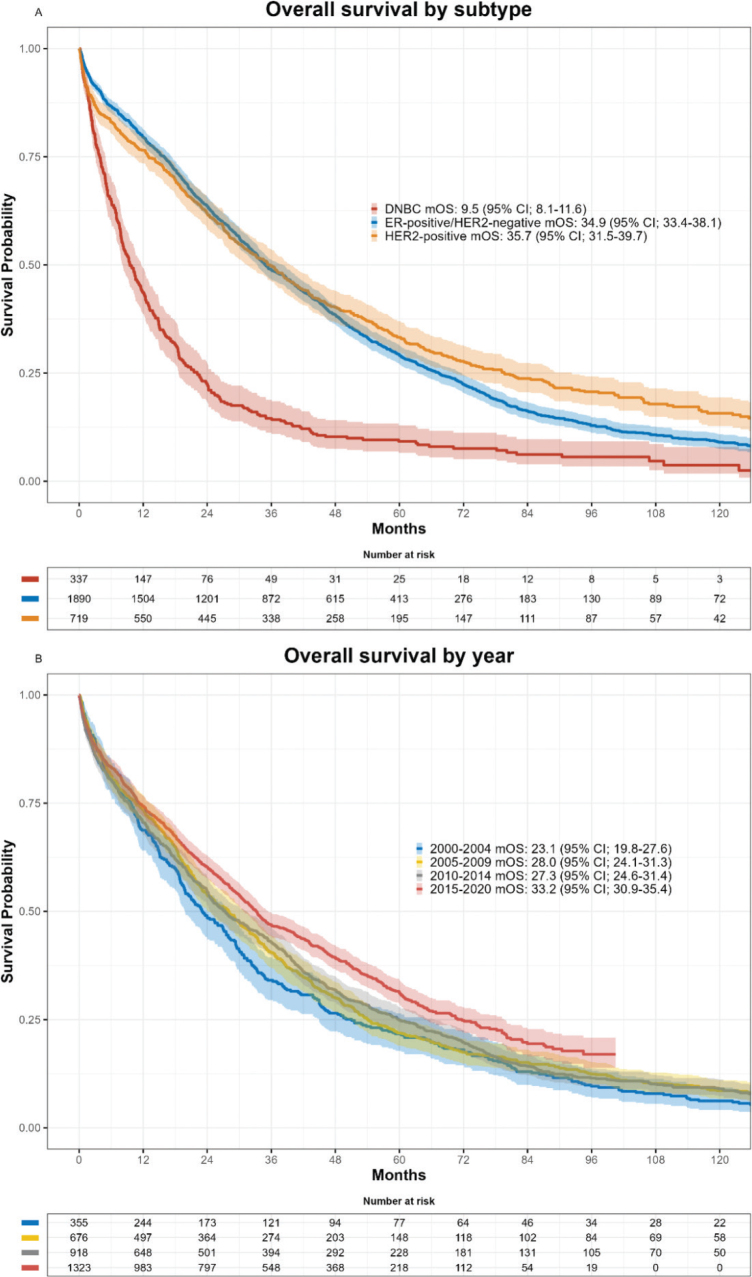
Kaplan-Meier curves for median overall survival by (A) subtype, (B) year of diagnosis. DNBC: Double-negative breast cancer, mOS: median overall survival, ER: estrogen receptor, HER2: human epidermal growth factor receptor 2.

In total, 2,752 patients died with an estimated median potential follow-up of 103.0 months (95% CI 99.1–108.0). The median OS was 29.3 months (95% CI; 28.0–30.9), rising from 23.1 (95% CI; 19.8–27.6) in 2000–2004 to 33.2 (95% CI; 30.9–35.4) in 2015–2020 (*p* < 0.001) (Table 3 and Figure 2). The difference according to time periods was, in unadjusted analysis, statistically significant only for DNBC (*p* = 0.02) but not for HER2-positive or ER-positive/HER2-negative tumours (Table 3). However, when examining time continuously and unadjusted, a highly statistically significant difference was seen for all three subtypes (Table 4), with improvement in survival for patients with ER-positive/HER2-negative or HER2-positive disease, and a decline in survival for DNBC by year of diagnosis, and a significant heterogeneity (*p* = 0.0008).

**Table 3:** Median, 1, 5, and 10-year survival for all patients and by subtype and year of diagnosis.

	Median Survival (95% CI)	*P*	One year (95% CI)	Five years(95% CI)	Ten years(95% CI)
**All patients**	29.3 (28.0, 30.9)		73% (71%, 74%)	26% (25%, 28%)	9.5% (8.3%, 11%)
Year of diagnosis		<0.001			
2000–2004	23.1 (19.8, 27.6)		69% (64%, 74%)	22% (18%, 26%)	6.2% (4.1%, 9.3%)
2005–2009	28.0 (24.1, 31.3)		74% (70%, 77%)	22% (19%, 25%)	8.6% (6.7%, 11%)
2010–2014	27.3 (24.6, 31.4)		71% (68%, 74%)	25% (22%, 28%)	9.0% (7.3%, 11%)
2015–2020	33.2 (30.9, 35.4)		74% (72%, 77%)	31% (29%, 34%)	— (—, —)
**ER-pos/HER2-neg**	34.9 (33.5, 38.2)		80% (78%, 81%)	29% (27%, 32%)	9.0% (7.5%, 11%)
Year of diagnosis		0.09			
2000–2004	32.9 (29.4, 45.0)		85% (79%, 93%)	29% (22%, 39%)	5.8% (2.7%, 13%)
2005–2009	33.5 (30.3, 37.8)		80% (76%, 84%)	24% (20%, 29%)	9.4% (6.8%, 13%)
2010–2014	33.5 (28.4, 38.6)		77% (74%, 81%)	28% (24%, 32%)	8.2% (6.2%, 11%)
2015–2020	39.7 (34.3, 44.9)		80% (78%, 83%)	33% (30%, 37%)	— (—, —)
**HER2-positive**	35.7 (31.5, 39.7)		77% (73%, 80%)	33% (30%, 37%)	16% (13%, 19%)
Year of diagnosis		0.20			
2000–2004	28.4 (24.4, 45.3)		81% (72%, 91%)	28% (19%, 41%)	10% (5.2%, 21%)
2005–2009	30.3 (25.5, 42.9)		79% (72%, 85%)	29% (23%, 37%)	12% (7.8%, 18%)
2010–2014	38.1 (33.3, 43.7)		77% (72%, 83%)	32% (27%, 40%)	17% (12%, 23%)
2015–2020	39.0 (31.1, 47.8)		74% (69%, 79%)	38% (33%, 45%)	— (—, —)
**DNBC**	9.5 (8.1, 11.6)		44% (39%, 50%)	9.4% (6.7%, 13%)	3.8% (1.8%, 8.1%)
Year of diagnosis		0.02			
2000–2004	14.9 (6.1, 23.0)		60% (44%, 83%)	8.0% (2.1%, 30%)	4.0% (0.6%, 27%)
2005–2009	10.5 (6.7, 16.2)		48% (37%, 63%)	10% (4.8%, 22%)	3.4% (0.9%, 13%)
2010–2014	7.4 (6.5, 9.3)		32% (24%, 43%)	3.0% (1.0%, 9.1%)	0.0% (0.0%, 0.0%)
2015–2020	11.0 (9.1, 14.3)		47% (40%, 55%)	13% (8.7%, 20%)	— (—, —)
**Unknown IHC**			52% (47%, 58%)	12% (8.6%, 16%)	4.0% (2.3%, 6.8%)
Year of diagnosis		0.057			
2000–2004	14.2 (11.1, 19.9)		54% (47%, 63%)	16% (11%, 23%)	5.0% (2.5%, 9.8%)
2005–2009	16.5 (11.3, 22.0)		56% (47%, 67%)	9.2% (4.9%, 17%)	3.1% (1.0%, 9.3%)
2010–2014	9.1 (5.4, 22.7)		44% (32%, 60%)	6.0% (2.0%, 18%)	4.0% (1.0%, 16%)
2015–2020	7.6 (0.7, 34.4)		39% (22%, 69%)	0% (0%, 0%)	— (—, —)

ER: Estrogen receptor; HER2: human epidermal growth factor receptor 2; DNBC: Double-negative breast cancer; IHC: immunohistochemistry.

**Table 4:** Uni- and multivariable Cox regression model for overall survival.

	Univariable			Multivariable		
	HR	95% CI	*P*	HR	95% CI	*P*
**Age×**	1.10	1.09, 1.12	<0.001	1.10	1.08, 1.12	<0.001
Age, time-dependent*	0.98	0.97, 0.99		0.98	0.97, 0.99	
**Year of diagnosisπ**						
ER-pos	0.98	0.97, 0.98	<0.001	0.98	0.96, 0.99	<0.001
DNBC	1.07	1.06,1.07	<0.001	0.99	0.97, 1.01	0.48
HER2-pos	0.98	0.97, 0.98	<0.001	0.97	0.96, 0.99	0.001
**Immunohistochemistry subtype**						
ER-pos/HER2-neg	Ref			Ref		
DNBC *year 0–2*	2.83	2.47, 3.24	<0.001	2.72	1.93, 3.84	0.001
DNBC *year 2+*	1.22	0.94, 1.60		0.96	0.65, 1.42	
HER2-positive	0.77	0.70, 0.84		1.00	0.78, 1.28	
HER2, time-dependent*	0.92	0.87, 0.98		0.83	0.78, 0.88	
**Charlson Comorbidity Index**						
0	Ref			Ref		
1–2	1.33	1.22, 1.46	<0.001	1.16	1.06, 1.27	<0.001
3	1.90	1.61, 2.23		1.50	1.28, 1.78	
CCI 3, time-dependent*	0.83	0.75, 0.90		0.89	0.81, 0.98	
**Visceral disease**						
No	Ref			Ref		
Yes	1.43	1.32, 1.55	<0.001	1.44	1.33, 1.56	<0.001
Visceral, time-dependent*	0.80	0.76, 0.85		0.83	0.78, 0.87	

HR: Hazard ratio; CI: Confidence interval; ER: estrogen receptor; HER2: human epidermal growth factor receptor 2; DNBC: Double-negative breast cancer.

× Continuous with 5-year increments,

* Each year reduces the estimate accordingly. Modelled with a log time-dependency due to lack of proportionality. ^π^Hazard per year of diagnosis for each IHC subtype.

*P*-values for the overall effect of age, IHC subtype, CCI, and visceral disease.

The association between OS and IHC subtype, age, year of diagnosis, comorbidity and visceral disease was investigated in a multivariable Cox regression model (Table 4). Age was associated with a decreased survival probability (HR; 1.10 95% CI, 1.08 to 1.12) as with CCI 1–2 (HR; 1.16 95% CI, 1.06 to 1.27) and CCI 3 (HR; 1.50 95% CI, 1.28 to 1.78), and visceral disease (HR; 1.44 95% CI, 1.33 to 1.56. These effects diminish over time (Table 4). Furthermore, DNBC was associated with a higher mortality (HR: 2.72 95% CI, 1.93 to 3.84) within the first 2 years compared to patients with ER-positive/HER-negative disease. Patients with HER2-positive disease fared equivalent to patients with ER-positive/HER2-negative disease (HR; 1.00 95% CI, 0.78 to 1.28), but with an improvement over time. In the multivariable model, the year of diagnosis remained significant with improved survival by increasing year for both ER-positive/HER-negative and HER2-positive subtypes, but not for DNBC; test for heterogeneity *p* = 0.01.

## Discussion

In a setting with population-based and nationwide registration of breast cancer, we observed a more than threefold increase in the incidence of pMBC over two decades. This apparent increase is, however, largely due to the changes in registration practice and stage migration, while it only, to a lesser degree, reflects an actual increase in pMBC. The increase in pMBC was significantly less pronounced among those aged 50 to 69 years, where screening was gradually implemented compared with those younger or older. Although the proportion of those aged 50 to 69 years was reduced from 55 to 34%, the absolute number more than doubled. Nationwide screening was gradually implemented during the study period, but only 41 of the pMBCs were screening-detected, while 88 were interval cancers. Considering the slower rise in incidence, our results do not rule out a positive effect of screening, for example prevention of pMBC by inverse stage migration [28].

Over the recent two decades, the incidence of pMBC increased sixfold among women aged 70 years or older, and this may be conditioned by more complete coding of surgery and pathology. Furthermore, staging practice has changed considerably. Where only 6% had an FDG-PET/CT or a CT combined with a bone evaluation (bone scintigraphy or MRI of the spine) from 2000 to 2004, that share increased to 65% for patients diagnosed with pMBC from 2015 to 2020. Still, more than a third of patients were insufficiently staged, even in the most recent period (Figure 1D). Besides the introduction of screening, changes in coding practices, and stage migration, other factors as well as yet unidentified factors, may have impacted the increased incidence observed in pMBC. This may seriously compromise the interpretation of the temporal evolution of subtype distribution, survival rates, and clinical factors for patients with pMBC.

The fact that 10% of patients had insufficient pathological assessment and could not be classified to an IHC subtype further hinders any meaningful subgroup analysis for the identified pMBC patients. Unfortunately, we do not know the reasons why these patients were not HER2-scored, as this was not coded. Furthermore, coding practice in the utilised registries also impacts our results, for example before 2004, it was not compulsory to report imaging to the NPR). Likewise, conventional radiotherapy was supplied by the NPR, where indication and target organ was not reported.

A rather large proportion of patients were not treated according to general guidelines. For instance, more than 20% of patients with DNBC, ER-positive, and HER2-positive disease did not receive chemotherapy, endocrine therapy, or HER2-targeted treatment in the first line, respectively. Furthermore, taxanes and anthracycline were gradually less frequently prescribed as first-line chemotherapy despite being the recommended choices for pMBC throughout the years. The use of surgery within 1 year of diagnosis decline significantly with time, reflecting emerging inconsistent data from randomised trial and the use of more sensitive staging procedures [29]. Unfortunately, we are unable to clarify to what extent comorbidities, shared decision-making, and changes in clinical thinking explains the observed deviations from guidelines.

It is impossible to determine whether the observed survival improvements over the years stem from an enhanced treatment regimen or that historically only symptomatic pMBC cases were diagnosed upfront, while those with subclinical metastases were identified later and thus not registered as pMBC. Regardless of the credibility of the possible improvement implied by the current result, patients with med pMBC continue to face a challenging prognosis, with a median OS of 33 months.

The strength of this study lies in its national perspective, which limits selection bias and has allowed us to identify a large, unselected group of patients with pMBC. Furthermore, with personal identification numbers, we have secured complete follow-up and linkage across several registers to highlight changes in practices for pMBC.

Other national registries have likewise examined the implications of changes in coding and classification practices and found fluctuations in the incidence of pMBC attributed to the introduction of TNM classification, screening, neoadjuvant therapy, and lymph node staging [30, 31]. Other studies that did not focus on stage migration, screening, or other confounders have seen divergent results with both decreasing, stable, and increasing incidence over time but a consistent increase in survival across time [32–35].

## Conclusion

The introduction of breast cancer screening, improved staging and coding practice have led to a substantial rise in the detection of pMBC between 2000 and 2020 in Denmark. The scope of these issues renders conclusions based on temporal shifts in survival rates unreliable.

## Ethics

The study was approved by DBCG’s oncological committee, the Capital Region’s research and the Capital Region’s Center for Health (R-22036280). This corresponds to informed consent for involved patients by Danish law.

## Competing interests

TB: Institutional grants from: Pfizer, Astra Zeneca, Novartis, Samsung Bioepis, Seattle Genetics, Merck, Eli Lilly and Daiichi Sankyo. Advisory board: Novartis. Travel: Daiichi Sankyo

MJ: Advisory board, Novartis.

MR: Advisory board: Merck, Talks: Merck, GSK. Institutional grant: Astra Zeneca

CTA, IK, LS, MV: None

AK: Institutional grants from Pfizer, AstraZeneca, Merck, Eli Lilly, Seattle Genetics, Roche, Novartis. Personal grants from Astra Zeneca (travel + advisory board), MSD (travel), Daiichi Sankyo (advisory Board), Novartis (advisory Board), Seagen (advisory Board), Gilead (advisory Board)

BE: Institutional grants from: Astra Zeneca, Daiichi Sankyo, Eli Lilly, Merck, Novartis, and Pfizer; Advisory board: Eli Lilly and Medac; Travel: Daiichi Sankyo and Merck

## Data availability

Data used to generate figures, tables, and supplementary tables and figures in the published article are not publicly available due to institutional restrictions. The dataset can be made available to qualified researchers through application to the Danish Breast Cancer Group.

## Author contributions

TB: Conceptualisation, methodology, investigation, data curation, formal statistical analysis, writing – original draft, writing – review and editing, visualisation

MJ: Conceptualisation, methodology, investigation, formal statistical analysis, writing – review and editing

MR: Conceptualisation, methodology, writing – review and editing

CTA: Data curation, writing – review and editing

IK: Data curation, writing – review and editing

LS: Data curation, writing – review and editing

MV: Data curation, writing – review and editing

AK: Conceptualisation, methodology, investigation, writing – review and editing

BE: Conceptualisation, methodology, investigation, writing – review and editing

## Supplementary Material

Incidence and survival of primary metastatic breast cancer in Denmark: implication of breast cancer screening, classification, and staging practice
