# Supplementary material for: Incidence and survival of primary metastatic breast cancer in Denmark: implication of breast cancer screening, classification, and staging practice
Source: Acta Oncol. 2024 May 6;63:37270. doi: 10.2340/1651-226X.2023.37270 (PMC11332488; doi:10.2340/1651-226X.2023.37270)
Supplement: Incidence and survival of primary metastatic breast cancer in Denmark: implication of breast cancer screening, classification, and staging practice [file AO-63-37270-s1.pdf]

Supplementary material has been published as submitted. It has not been copyedited or typeset by Acta Oncologica.

Supplementary Table 1 – age by subtype for all patients with primary metastatic breast cancer and a known immunohistochemical subtype.

**Age Group**	**DNBC**, N = 337	**ER-pos/HER2-neg**, N = 1,890	**HER2-pos**, N = 719
<40	26 (22%)	37 (31%)	57 (47%)
40-49	30 (9.8%)	181 (59%)	95 (31%)
50-59	59 (12%)	306 (60%)	143 (28%)
60-69	82 (11%)	480 (65%)	176 (24%)
70-79	82 (9.9%)	580 (70%)	165 (20%)
80+	58 (13%)	306 (68%)	83 (19%)

Row percentage.
DNBC: double-negative breast cancer, HER2: human epidermal growth factor receptor 2, ER: estrogen receptor

Supplementary Table 2 – age by ER and HER2 for all patients with primary metastatic breast cancer.

	**Negative (HER2)**		**Positive (HER2)**		**NA (HER2)**	
**Age Group**	**ER-Negative**, N = 337	**ER-Positive**, N = 1,890	**ER-Negative**, N = 299	**ER-Positive**, N = 419	**ER-Negative**, N = 39	**ER-Positive**, N = 275
<40	26 (21%)	37 (30%)	21 (17%)	36 (30%)	1 (0.8%)	1 (0.8%)
40-49	30 (9.4%)	181 (57%)	43 (14%)	52 (16%)	5 (1.6%)	7 (2.2%)
50-59	59 (11%)	306 (57%)	59 (11%)	84 (16%)	7 (1.3%)	21 (3.9%)
60-69	82 (10%)	480 (60%)	73 (9.1%)	102 (13%)	8 (1.0%)	57 (7.1%)
70-79	82 (8.8%)	580 (62%)	72 (7.7%)	93 (10%)	12 (1.3%)	93 (10%)
80+	58 (11%)	306 (56%)	31 (5.6%)	52 (9.5%)	6 (1.1%)	96 (17%)

Row percentage.
HER2: human epidermal growth factor receptor 2, ER: estrogen receptor
13 patients not shown due to unknown ER-status; 1 patient was HER2 negative, ER unknown, 1 HER2 positive, ER unknown and 11 were HER2 and ER unknown.
